# Prevalence of the Crayfish Plague Pathogen *Aphanomyces astaci* in Populations of the Signal Crayfish *Pacifastacus leniusculus* in France: Evaluating the Threat to Native Crayfish

**DOI:** 10.1371/journal.pone.0070157

**Published:** 2013-07-23

**Authors:** Lenka Filipová, Adam Petrusek, Klára Matasová, Carine Delaunay, Frédéric Grandjean

**Affiliations:** 1 Department of Ecology, Faculty of Science, Charles University in Prague, Prague, Czech Republic; 2 Laboratoire Ecologie et Biologie des Interactions, Equipe « Ecologie, Evolution, Symbiose », UMR 7267 CNRS, Université de Poitiers, Poitiers, France; University of Toronto, Canada

## Abstract

*Aphanomyces astaci*, the crayfish plague pathogen, first appeared in Europe in the mid-19^th^ century and is still responsible for mass mortalities of native European crayfish. The spread of this parasite across the continent is especially facilitated by invasive North American crayfish species that serve as its reservoir. In France, multiple cases of native crayfish mortalities have been suggested to be connected with the presence of the signal crayfish *Pacifastacus leniusculus*, which is highly abundant in the country. It shares similar habitats as the native white-clawed crayfish *Austropotamobius pallipes* and, when infected, the signal crayfish might therefore easily transmit the pathogen to the native species. We investigated the prevalence of *A. astaci* in French signal crayfish populations to evaluate the danger they represent to local populations of native crayfish. Over 500 individuals of *Pacifastacus leniusculus* from 45 French populations were analysed, plus several additional individuals of other non-indigenous crayfish species *Orconectes limosus*, *O. immunis* and *Procambarus clarkii*. Altogether, 20% of analysed signal crayfish tested positive for *Aphanomyces astaci*, and the pathogen was detected in more than half of the studied populations. Local prevalence varied significantly, ranging from 0% up to 80%, but wide confidence intervals suggest that the number of populations infected by *A. astaci* may be even higher than our results show. Analysis of several individuals of other introduced species revealed infections among two of these, *O. immunis* and *P. clarkii*. Our results confirm that the widespread signal crayfish serves as a key reservoir of *Aphanomyces astaci* in France and therefore represents a serious danger to native crayfish species, especially the white-clawed crayfish. The prevalence in other non-indigenous crayfish should also be investigated as they likely contribute to pathogen transmission in the country.

## Introduction

For more than 150 years, native crayfish in Europe have been decimated by the crayfish plague, a disease caused by the oomycete *Aphanomyces astaci*. The first presumed European outbreak of crayfish plague was recorded in 1859 in northern Italy, and another focus of the disease appeared in France in 1874 at Plateau des Langres [1], [2]. In the following decades, the pathogen continued to spread to other European countries [1], [2], [3], [4]. At present, the known carriers of the pathogen in Europe are three North American crayfish species that were introduced to the continent before 1975, the so-called “old” non-indigenous crayfish species: the spiny-cheek crayfish *Orconectes limosus*, the signal crayfish *Pacifastacus leniusculus*, and the red swamp crayfish *Procambarus clarkii* [5]. Their presence in European waters facilitates the persistence and spread of the parasite, and further contributes to mortalities of native crayfish [4], [5], [6], [7], [8].

The largest mass mortalities of native crayfish in France took place between the 1870s and 1912 [9], [10], but after a relatively calm period up until the 1980s, new outbreaks of crayfish plague have again been reported [10], [11], [12]. Some of these mortalities of native species were suspected to be connected with the presence of the invasive signal crayfish *Pacifastacus leniusculus* [12], [13], [14], [15], [16]. The link between the presence of this species and crayfish plague spread is also apparent in other European countries [6], [17], [18].

The signal crayfish was introduced to France in 1972 from Sweden, and in 1974 more individuals were brought directly from North America (Lake Tahoe and Lake Donner in California); this was followed by numerous secondary introductions [19], [20]. The species is now widely distributed in France [3], [4], [19], [21], [22], [23]. In 2006, it was estimated to be present at about 1000 sites in 73 out of 96 French departments [23] representing all 22 administrative regions of continental France. Although several other non-indigenous crayfish species are found in France, *Pacifastacus leniusculus* represents the largest threat to native species, particularly to the white-clawed crayfish *Austropotamobius pallipes* [23]. Signal crayfish may colonise similar habitats in the headwaters of rivers and therefore easily come into contact with *A. pallipes* populations [24]. This facilitates the transmission of the pathogen to the native species if the invasive one is infected.

**Figure 1:**
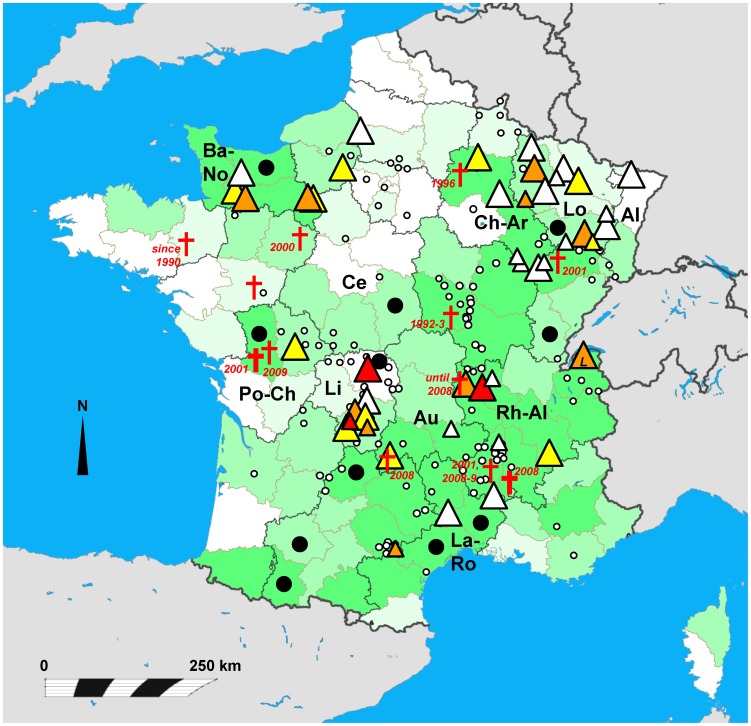
Map of France with administrative division to regions (dark-bordered areas) and departments within them (light-bordered areas), showing the distribution of the invasive signal crayfish *Pacifastacus leniusculus* (small empty circles; based on [4], [19], [21], [44]) and approximate location of analysed populations (triangles), and the recent status of the native white-clawed crayfish *Austropotamobius pallipes* (green shading) and reported cases of its mass mortalities (red crosses and black dots). Distribution *of A. pallipes* is based on a 2006 survey [23] (white: no known population in a department, pale green: 1–5, medium: 6–25, dark: 25–150 populations). Red crosses with years indicate mass mortalities most likely caused by crayfish plague reported since the 1990s ([10], [12], [13], [14], [15], [45], [46]; T. Duperray and T. Pantarotto, pers. comm.); additional mortalities ascribed to crayfish plague were reported between 2001 and 2005 from departments marked by black dots (according to [23]). Bold crosses mark outbreaks in which *A. astaci* has been confirmed by molecular detection and genotyped (see Discussion). The prevalence of *Aphanomyces astaci* in sampled signal crayfish populations is expressed by colour: no reliable detection of the pathogen (white triangles), low prevalence (1–30%, yellow), medium prevalence (31–60%, orange) and high prevalence (61–100%, red triangles); the number of analysed individuals in the respective population is indicated by symbol size (small triangle: <10 individuals, large triangle: 10+ individuals). Regions discussed in the text are abbreviated: Al – Alsace, Au – Auvergne, Ba-No – Basse-Normandie, Ce – Centre, Ch-Ar – Champagne-Ardenne, La-Ro – Languedoc-Rousillon, Li – Limousin, Lo – Lorraine, Po-Ch – Poitou-Charentes, Rh-Al – Rhône-Alpes; Lake Geneva (Lac Léman) is marked by L within the triangle. The distance scale depends on latitude (top: 51.5°N, bottom: 41°N) and reflects the map projection.

The white-clawed crayfish *Austropotamobius pallipes* is the most abundant indigenous European crayfish in France. In 2006, it was still found at over 2200 sites in 76 out of 96 departments, i.e., in all but one administrative region of continental France (Fig. 1; [23]). However, the number of its populations has significantly decreased recently and is substantially lower than that of alien crayfish [23]. The crayfish plague is one of the major factors that contribute to the decline of *A. pallipes* in France [24]; eighty-nine per cent of crayfish mass mortalities recorded in France between the years 2001 and 2006 affected *Austropotamobius pallipes*, while only the remaining 11% of mortalities concerned other species [23].

Despite the substantial impact the crayfish plague has on native crayfish species, reliable information on the distribution of its pathogen *A. astaci* in European waters is rather scattered. For many years, mortalities of native crayfish, if noticed, suggested the presence of the disease. However, the identity of the pathogen was often assumed rather than confirmed, due to difficulties with its cultivation and ambiguous morphological characteristics [25], [26]. Recently, several molecular methods for the detection of *Aphanomyces astaci* have been developed that do not require cultivation [27], [28], [29], [30], [31]. Very low quantities of the pathogen DNA in the sample are detectable by some of these methods, enabling large-scale screening of populations of invasive crayfish from different European countries.

Interestingly, recent molecular studies have suggested that some populations of invasive crayfish in Europe might not be infected by *A. astaci* or its prevalence is very low, and in those confirmed to host the pathogen the prevalence and load may vary substantially [32], [33]. So far, the largest datasets have been obtained by Kozubíková *et al.* [33], [34], who studied crayfish plague prevalence in more than 300 individuals of *Orconectes limosus* and more than 100 individuals of *Pacifastacus leniusculus,* representing 29 Central European populations. In other studies focusing on the presence of this pathogen in invasive crayfish in Europe, only one or a few populations were analysed (e.g., [32], [35], [36], [37]).

No data on the prevalence of the crayfish plague pathogen in non-indigenous crayfish populations in France have been available so far. Therefore, we tested in the present study numerous French populations of *Pacifastacus leniusculus* as well as a few individuals of other non-indigenous crayfish species for the presence of *Aphanomyces astaci*. A quantitative TaqMan minor groove binder (MGB) real-time polymerase chain reaction (PCR) designed by Vrålstad *et al.* [30] was chosen for our analyses, as it has been shown to be the most sensitive of the available detection methods, and highly specific to this pathogen species [33], [38].

Our aim was to evaluate the threat that signal crayfish populations in different regions of France represent to native crayfish species. We specifically focused on this invasive crayfish species because its distribution and habitat preferences most overlap with native crayfish in France [23]. Data on the prevalence of *A. astaci* and infection intensities expressed as pathogen levels in the hosts’ tissues may then allow targeting the most infected invasive crayfish populations for potential future eradication trials, and pinpointing native crayfish populations that are at highest risk and could be translocated to safer areas, i.e., “ark sites” [39]. Thus, data on the distribution and prevalence of the crayfish plague pathogen in the country may contribute to improving the efficiency of conservation management of indigenous crayfish species, in particular *Austropotamobius pallipes*.

## Methods

### Ethics Statement

All experimental procedures and animal manipulations, as well as field sampling, conformed to French law. The analysed material was provided by the French National Agency for Water and Aquatic Environments (ONEMA), which is entitled to collect samples in French watercourses, including those located on privately owned land. No additional permits were required for the described field studies. The project did not involve work with endangered or protected species, apart from samples of dead individuals collected from mass mortalities of the native crayfish species *Austropotamobius pallipes*.

### Sampling, DNA Isolation and Real-time PCR

In total, 513 signal crayfish *Pacifastacus leniusculus* individuals were sampled from 45 localities in France (Table 1, Fig. 1) by hand or by electrofishing. Although these sites represent only a small fraction of invaded sites in the country (see [23]), they cover a substantial part of most invaded regions. Most individuals came from running waters, especially brooks and several rivers. In addition, several individuals of other non-indigenous species were also analysed. These were 19 individuals of *Orconectes limosus* from two populations, seven individuals of *O. immunis* from one population, and two individuals of *Procambarus clarkii* from one population (Table 1). The sampled populations, their characteristics, and the numbers of analysed individuals are summarised in Table 1.

**Table 1:** Results of *Aphanomyces astaci* detection in 45 analysed French populations of the signal crayfish *Pacifastacus leniusculus* and four additional populations of other non-indigenous crayfish species (*Procambarus clarkii*, *Orconectes limosus* and *O. immunis*).

Sp.	Locality	Region	River basin	Coordinates	Sampling date	No. inf./analysed	Prevalence	(95% CI)	Agent level
***Pacifastacus leniusculus***									
	river La Cère, Sansac de Marmiesse	Auvergne	Dordogne	44°53′N, 2°22′E	6 Sep 2010	2/10	20%	(3–56%)	A2
	brook La Senouire, St. Pal de Senouire	Auvergne	Loire	45°15′N, 3°39′E	26 Jul 2010	0/5	0%	(0–64%)	–
	brook du Parc, Mesnil-Auzouf	Basse-Normandie	coastal brook	48°59′N, 0°43′W	31 Mar 2010	0/18	0%	(0–26%)	–
	brook Varenne, Saint-Bômer-les-Forges	Basse-Normandie	Loire	48°37′N, 0°36′W	2 Sep 2009	8/14	57%	(29–82%)	A2(6), A3(2)
	brook Egrenne, Beauchêne	Basse-Normandie	Loire	48°41′N, 0°45′W	2 Sep 2009	4/15	27%	(8–55%)	A2
	brook Sarthon, St. Denis-sur-Sarthon	Basse-Normandie	Loire	48°27N, 0°03′W	Oct 2009	4/14	29%	(8–58%)	A2
	brook Sarthon, Rouperroux	Basse-Normandie	Loire	48°32N, 0°05′W	Oct 2009	7/16	44%	(20–70%)	A2(6), A3(1)
	brook Côte Saint-Gilles, St. Aubin sur Gaillon	Haute-Normandie	Seine	49°08′N, 1°21′E	2 nov 2009	1/12	8%	(0–38%)	A2
	brook Mesangueville, Dampierre-en-Bray	Haute-Normandie	Seine	49°32′N, 1°40′E	17 Sep 2009	0/10	0%	(0–41%)	–
	brook Le Vannon, Genévrieres	Champagne-Ardenne	Saône	47°42′N, 5°36′E	16–17 Sep 2009	0/14	0%	(0–32%)	–
	brook Petits Crots, Poinson-les-Fayl	Champagne-Ardenne	Saône	47°45′N, 5°35′E	24 Sep 2009	0/5	0%	(0–64%)	–
	brook Ource, Colmier-le-Bas	Champagne-Ardenne	Seine	47°46′N, 4°57′E	18 Sep 2009	0/5	0%	(0–64%)	–
	brook Aube, Rouvres sur Aube	Champagne-Ardenne	Seine	47°51′N, 5°00′E	2 Sep 2009	0/5	0%	(0–64%)	–
	brook Bruxenelle, Plichancourt	Champagne-Ardenne	Seine	48°45′N, 4°40′E	3 Sep 2009	0/22	0%	(0–22%)	–
	river La Vesle, Prunay	Champagne-Ardenne	Seine	49°11′N, 4°11′E	2 Sep 2009	5/17	29%	(10–56%)	A2
	brook La Foux, Lanuejols	Languedoc-Rousillon	Garonne	44°07′N, 3°25′E	28 May 2009	0/10	0%	(0–41%)	–
	brook Moze, St. Julien-de-Peyrolas	Languedoc-Rousillon	Rhône	44°17′N, 4°34′E	11 Nov 2009	0/10	0%	(0–41%)	–
	brook La Mayne, Concèze	Limousin	Dordogne	45°21′N, 1°21′E	2 Aug 2010	1/12	8%	(0–38%)	A2
	brook La Vézère, Uzerche	Limousin	Dordogne	45°25′N, 1°34′E	3 Aug 2010	4/5	80%	(28–99%)	A2(3), A3(1)
	brook La Douyge, St. Augustin	Limousin	Dordogne	45°25′N, 1°51′E	29 Jul 2010	4/9	44%	(14–79%)	A2
	brook La Maulde, St. Martin Chateau	Limousin	Loire	45°51′N, 1°49′E	6 Jul 2010	0/12	0%	(0–36%)	–
	brook Cherpont, Sainte-Feyre	Limousin	Loire	46°09′N, 1°57′E	4 Jun 2009	11/15	73%	(45–95%)	A2(7), A3(4)
	brook La Petite Briance, St. Germain les Belles	Limousin	Loire	45°37′N, 1°30′E	3 Jun 2010	3/9	33%	(7–70%)	A2(2), A3(1)
	brook La Grande Briance, Croiselle sur Briance	Limousin	Loire	45°36′N, 1°36′E	29 Jul 2010	2/14	14%	(2–43%)	A2
	brook Longeau, Allamont	Lorraine	Rhine	49°07′N, 5°48′E	25 Sep 2009	0/5	0%	(0–64%)	–
	river Orne, Hatrize	Lorraine	Rhine	49°12′N, 5°55′E	26 Aug 2009	0/15	0%	(0–30%)	–
	brook Othain, Petit-Failly	Lorraine	Meuse	49°26′N, 5°29′E	27 Aug 2009	0/18	0%	(0–26%)	-
	pond Claveau, Cirey-sur-Vezouze	Lorraine	Rhine	48°36′N, 6°58′E	4 Oct 2010	0/8	0%	(0–48%)	–
	brook Seigneulle, St. Maurice	Lorraine	Rhine	49°01′N, 5°41′E	1 Oct 2009	0/15	0%	(0–30%)	–
	brook Zinzel du Nord, Baerenthal	Lorraine	Rhine	48°59′N, 7°30′E	3 Sep 2009	0/13	0%	(0–34%)	–
	brook Nied, Aube	Lorraine	Rhine	49°01′N, 6°20′E	4 Sep 2009	1/10	10%	(0–45%)	A3
	pond de la Prairie du Vouau, St. Nabord	Lorraine	Rhine	48°03′N, 6°36′E	15 Aug 2009	1/5	20%	(1–72%)	A2
	brook Saône, Vioménil	Lorraine	Saône	48°05′N, 6°10′E	26 Aug 2009	0/5	0%	(0–64%)	–
	brook des Noires Faignes, Aneuménil	Lorraine	Rhine	48°06′N, 6°32′E	25 Aug 2009	5/15	33%	(12–62%)	A2(2), A3(3)
	river Meurthe, St. Michel sur Meurthe	Lorraine	Rhine	48°19′N, 6°55′E	26 Aug 2009	0/12	0%	(0–36%)	–
	brook Ezrule, Chaumont-sur-Aire	Lorraine	Seine	48°56′N, 5°15′E	1 Oct 2009	2/5	40%	(5–85%)	A2(1), A3(1)
	brook Orne, Ornel	Lorraine	Rhine	49°15′N, 5°37′E	24 Sep 2009	8/15	53%	(27–79%)	A2
	river Thoré, St. Amans-Soult	Midi-Pyrénées	Garonne	43°29′N, 2°29′E	1 Oct 2009	3/9	33%	(7–70%)	A2(2), A3(1)
	brook Miosson, Bertandinière, Smarves	Poitou-Charentes	Loire	46°31′N, 0°22′E	18 Jun 2010	3/15	20%	(4–48%)	A2(1), A3(1), A4(1)
	brook Grozon, St. Barthélémy-Grozon	Rhône-Alpes	Rhône	44°59′N, 4°37′E	27 Aug 2009	0/4	0%	(0–72%)	–
	lake Geneva (Léman), Thonon-les-Bains	Rhône-Alpes	Rhône	46°23′N, 6°29′E	Jul 2009	5/16	31%	(11–59%)	A2(4), A3(1)
	lake Laffrey, La Bergogne	Rhône-Alpes	Isère	45°00′N, 5°47′E	Sep 2009	1/13	8%	(0–36%)	A2
	brook Charpasonne, Panissières	Rhône-Alpes	Loire	45°47′N, 4°20′E	2 Jul 2009	10/14	71%	(42–92%)	A2(3), A3(6), A4(1)
	brook Aix, Grézolles	Rhône-Alpes	Loire	45°51′N, 3°57′E	26 Aug 2008	8/20	40%	(19–64%)	A2(3), A3(5)
	river Azergues, Ternand	Rhône-Alpes	Saône	45°57′N, 4°32′E	3 Oct 2009	0/3	0%	(0–81%)	–
**other species**									
***Procambarus clarkia***									
	pond La Chaume, Rosnay	Centre	Loire	46°42′N, 1°13′E	4 Mar 2011	1/2	50%	(1–99%)	A2
***Orconectes limosus***									
	pond Barineau, Rosnay	Centre	Loire	46°42′N, 1°13′E	4 Mar 2011	0/3	0%	(0–81%)	–
	brook Ramiers, Vernoux en Vivarais	Rhône-Alpes	Rhône	44°54′N, 4°39′E	19 May 2009	0/16	0%	(0–29%)	–
***Orconectes immunis***									
	brook Reipertswiller, Rothbach	Alsace	Rhine	48°56′N, 7°30′E	8 Nov 2010	2/7	29%	(4–71%)	A2

Number of analysed and infected (inf.) individuals per population, prevalence of the crayfish plague pathogen *Aphanomyces astaci* (with 95% confidence intervals), and agent level in infected individuals are given for each population. Numbers in brackets in the last column indicate the number of infected specimens with that particular agent level (individuals with agent levels A0 and A1 are not considered). Where no number is provided, the same agent level was detected in all infected individuals from the population.

Captured crayfish were stored in 96% ethanol. Tissue from one half of the soft abdominal cuticle and one uropod (body parts most suitable for the detection of *A. astaci*, [36]) was dissected from each crayfish using sterile tools. While processing each individual, we noted the presence of black melanised spots, as possible visual symptoms of immune reaction to pathogens [40] and a characteristic used in the past to assess the infection status in signal crayfish (e.g., [41], [42]). Dissected tissues from each individual were collected in a single 1.5 ml tube, dried and stored in a deep freezer at −80°C. Before further processing, 360 µl of Buffer ATL from the DNeasy tissue kit (Qiagen) was added to the thawed dissected material. The mixture was then crushed by one scoop (ca 50 µl) of stainless steel beads (1.6 mm diameter) using a BBX24B Bullet Blender (Next Advance) for 10 min at maximum speed. DNA extractions from the crushed cuticle then followed the rest of the spin-column protocol of the DNeasy tissue kit, in double volume (i.e., with 40 µl of the proteinase K solution and 400 µl of Buffer AL).

The isolated material was then tested for the presence of *Aphanomyces astaci* by the quantitative TaqMan MGB real-time PCR [30], using a LightCycler® 480 Instrument (Roche). A 59 bp fragment of the internal transcribed spacer (ITS) region of *A. astaci* nuclear rDNA was amplified using the primers AphAstITS-39F (5′-AAG GCT TGT GCT GGG ATG TT-3′) and AphAstITS-97R (5′-CTT CTT GCG AAA CCT TCT GCT A-3′), and quantified with the pathogen-specific TaqMan® minor groove binder (MGB) probe AphAstITS-60P (5′-6-FAM-TTC GGG ACG ACC C-MGBNFQ-3′). The 25 µl reaction volume consisted of a 2x Universal PCR Master Mix (Applied Biosystems), both primers (500 nM each), TaqMan MGB-Probe (200 nM), nuclease free water, and template DNA (around 20 ng/µl). The PCR program consisted of one cycle of 10 min at 95°C, 50 cycles of 15 sec at 95°C and 60 sec at 58°C, and one final cycle of 60 sec at 40°C. In each run, two replicates of four different standards were included that served as positive controls and ensured the comparability of different runs. The quantity of the pathogen DNA in these standards, expressed in PCR forming units (PFU), were 3×4^10^, 3×4^8^, 3×4^4^ and 3×4^2^, respectively [30]. Two negative controls (which remained negative in all runs) were included in each run to detect possible contamination. For each isolate, undiluted and a 10-fold diluted replicate were analysed to test for the impact of inhibition that might influence the efficiency of detection [30], [43]. When some effects of inhibition were occasionally detected (mostly in samples with low agent level), the PFU values were estimated as described in [33].

### Data Analysis

Based on their PFU values, samples were classified into semi-quantitative categories of pathogen load, ranging from A0 (no traces of *A. astaci* DNA) to A7 (extremely high amounts of *A. astaci* DNA in the sample), as proposed by Vrålstad *et al.* [30]. Only individuals with agent level A2 and higher were considered infected. Agent level A1 falls below the limit of detection of the method (corresponding to 5 PFU) and may not only indicate trace amounts of pathogen DNA, but also false positives or minor contamination during analyses. Therefore, agent level A1 should not be considered a confirmation of the presence of *A. astaci* in the sample [30], [33].

We estimated the prevalence of *A. astaci* in studied populations and its 95% confidence interval, using the function “epi.conf” included in the library epiR [47] for the statistical package R v. 3.0 [48]. Furthermore, we evaluated the relationship between the prevalence of *A. astaci* in tested populations and the pathogen load in infected individuals (with agent level A2 or higher, log-transferred) from each population by calculating the logistic regression with quasibinomial distribution of errors (due to overdispersion) by generalised linear model (GLM) in R. Potential outliers were identified using Cook’s distance and leverage of residuals, and the analysis was also repeated with the dataset excluding such populations.

## Results

We detected the presence of the crayfish plague pathogen in more than half of the studied signal crayfish populations across the whole country (Fig. 1). The number of *Aphanomyces astaci*-positive individuals in sampled populations, agent levels detected in infected specimens and the crayfish plague prevalence for each population are summarised in Table 1.

In total, 103 signal crayfish (20% of analysed individuals) from 24 populations (i.e., 53% out of 45 tested) were found to be infected (with agent level A2 or higher) (Table 1). The pathogen prevalence in samples from the studied populations was highly variable, ranging from 0% to 80%; however, due to often low number of individuals analysed per populations, the confidence intervals for the prevalence estimates remain wide (Table 1). Thus, lack of unambiguous detection of the pathogen at a particular site cannot be considered as an evidence for its absence from the population; substantially higher sample sizes are needed to test for such scenario [49].

In 322 signal crayfish, no traces of *Aphanomyces astaci* DNA were found (agent level A0), and 88 individuals were assigned to agent level A1 (i.e., a very weak signal not considered as positive pathogen detection). Most samples (73 individuals) that tested positive contained a low amount of pathogen DNA (agent level A2), while agent level A3 was found in 28 individuals and A4 in two individuals (Table 1). Black melanised spots were observed on the cuticle of 17 signal crayfish individuals from nine localities; however, *A. astaci* DNA was detected in only three of such crayfish from different localities: Sarthon, Lake Geneva (Lac Léman), and Charpasonne.

The distribution of *P. leniusculus* populations with the highest crayfish plague prevalence (over 50%) was rather scattered, as these were located in Limousin (central France; Li in Fig. 1), Rhône-Alpes (eastern France; Rh-Al), Basse-Normandie (northwestern France; Ba-No) and the Lorraine region (northeastern France; Lo) (Table 1, Fig. 1). In some regions, *A. astaci*-positive individuals were found in the majority of local populations, such as in Limousin (6 infected out of 7 analysed populations), Rhône-Alpes (4/6), and Basse-Normandie (4/5). On the other hand, relatively low numbers of infected individuals were found in most populations from the Lorraine region, where only 17 individuals from 141 analysed signal crayfish tested positive for *A. astaci* (in 5 out of 13 analysed populations), or in populations from Champagne-Ardenne (Ch-Ar) with five infected out of 68 tested individuals, coming from a single population where 6 individuals were analysed (Table 1). In Languedoc-Rousillon (La-Ro), none of the signal crayfish tested positive for the presence of *A. astaci*; however, only 20 individuals from two populations were analysed from this region.

**Figure 2:**
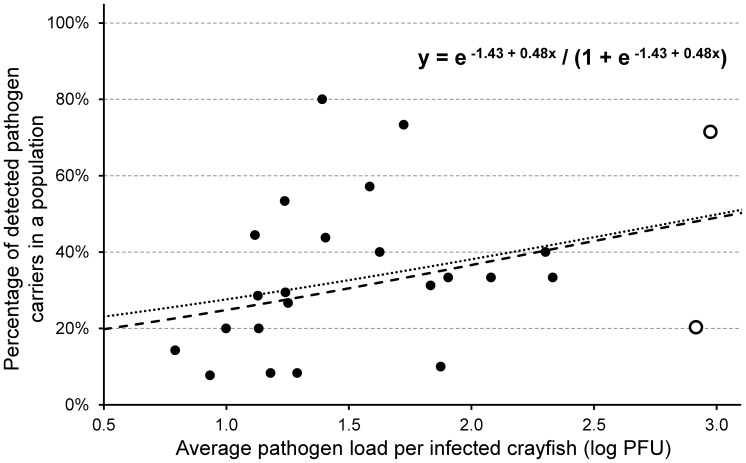
Relationship between the prevalence of *A.* *astaci* (in %; estimated as the proportion of individuals testing positive) in analysed French signal crayfish populations and the average pathogen load (expressed as log-transformed PFU-values) detected in infected individuals (with agent level A2 or higher) from each population. The equation characterising the model estimated by logistic regression (dashed line) is given in the upper right corner; removal of two outlier populations with highest average pathogen load (indicated by empty circles) did not change the model substantially (dotted line, equation not shown). Note that when a quasibinomial instead of binomial distribution of errors is used (due to overdispersion in data), the increasing trend is non-significant.

We observed a relatively weak trend of increase of *A. astaci* prevalence in signal crayfish populations with the average pathogen load per infected individuals from respective populations (Fig. 2); this increase was nevertheless not significant (GLM; df = 1, 23; p = 0.12). Based on criteria used to define outliers, we excluded two most influential points (populations with highest average pathogen load, shown in Fig. 2); their removal neither improved the significance of the model nor its general trend.

The real-time PCR analyses also confirmed the presence of *A. astaci* in two out of three other crayfish species analysed. None of the analysed individuals of *Orconectes limosus* from either of the two sampled populations (Rhône-Alpes and Centre regions) showed traces of the pathogen. However, two out of seven tested individuals (29%) of *Orconectes immunis* from Alsace tested positive, as did one of the two analysed individuals of *Procambarus clarkii* from the Centre region (Table 1).

## Discussion

Our results provide the first insight into the prevalence of the crayfish plague pathogen in invasive crayfish in France. We confirmed that the signal crayfish *Pacifastacus leniusculus* serves as an important reservoir of this disease in France, which strongly supports the notion of the key role of this invasive species in mortalities of native crayfish. We also showed independently on a recent study by Schrimpf et al. [50] that *Orconectes immunis*, a crayfish invader established only in the early 1990s [5], can be a carrier of *A. astaci*. This is the first of the “new” non-indigenous crayfish species in Europe, i.e., those introduced to the continent after 1980 [5], tested for this pathogen presence. Its positive infection status calls for investigating of other established North American crayfish species as well.

Our study confirms that a substantial proportion of signal crayfish from France carried *Aphanomyces astaci*. Despite the fact that the pathogen’s DNA was unambiguously detected in no more than 20% of analysed individuals, *A. astaci* presence was confirmed in 24 out of 45 studied host populations, representative for a substantial part of the invaded range in France. Furthermore, it may be assumed that the proportion of infected populations is substantially higher, given the wide confidence intervals for pathogen prevalence in individual populations. In Central European populations of this species (from the Czech Republic, Slovakia, and Hungary), Kozubíková *et al.* [33] also showed that a substantial proportion of analysed individuals tested positive for *A. astaci*, with 32 infected out of 153 analysed individuals, coming from 8 out of 9 sampled populations. In one population from Norway analysed by Vrålstad *et al.* [36], 38 out of 44 signal crayfish (86%) were infected; such high prevalence within a population was only rarely found in France. In contrast, no traces of the pathogen DNA were detected in 44 individuals from the river Alling Å in Denmark [51]. The number of infected individuals of another intensively studied invasive crayfish in Central Europe, *Orconectes limosus*, was found to be generally higher than in signal crayfish [33]: 116 out of 307 tested individuals (38%) were infected, coming from 16 out of 20 analysed populations [33]. Our results are also consistent with findings that crayfish infected by *A. astaci* may not show any macroscopic melanisation suggesting visually the presence of a pathogen [34], [35], and, on the contrary, that crayfish individuals (of various species) exhibiting strongly melanised spots may not test positive as *A. astaci* carriers [37], [52], [53].

The present results, although clearly demonstrating the widespread presence of the crayfish plague pathogen, should be interpreted carefully because the real prevalence of *A. astaci* is almost certainly underestimated in many cases. As only a small number of individuals could be tested from some sites, lack of detection of *A. astaci* cannot be interpreted as the absence of the pathogen from the population (see the 95% confidence intervals for the prevalence in Table 1). Providing conclusive evidence that some populations of potential *A. astaci* hosts (North American invasive crayfish) are likely to be uninfected requires much more intensive sampling effort [49]. Temporal fluctuations of the pathogen prevalence (or detectability) could also influence our results, as was shown for a Czech *Orconectes limosus* population studied over several seasons [54]. Thus, low *A. astaci* prevalence detected in some French signal crayfish populations does not mean that those pose a lower threat than other more infected populations of the same species.

Moreover, only certain parts of the crayfish cuticle (from the abdomen and uropod) were analysed, the isolation efficiency might have differed among samples, and just a part of DNA isolate was used in the real-time PCR. All these factors might further contribute to underestimations. However, as uropod tissue seems to be generally more infected than other parts of the crayfish body in signal crayfish [36], we assume that some pathogen DNA should have been present in samples from most infected crayfish individuals analysed in our study. Other characteristics and limits of this method (such as its specificity against other closely related species of *Aphanomyces*, or its sensitivity) are discussed in detail elsewhere [33], [38]. In general, however, the real-time PCR detection of *A. astaci* can be considered well validated [33], [38]. Thus, although our results are based on this method only, and we did not use alternative methods to confirm the pathogen (e.g., amplification and sequencing of a longer DNA fragment), we are convinced that the positive results indeed reflect the detection of *A. astaci*. Furthermore, we used some of the individuals from our study to test performance of newly developed microsatellite markers for this pathogen (F. Grandjean et al., in prep.), and the allele sizes obtained from infected French *P. leniusculus* matched perfectly those obtained from pure culture of *A. astaci* strain isolated from the same host species (F. Grandjean et al., unpubl. data). We consider this a convincing evidence that such crayfish individuals were infected by the same or very closely related strain of the pathogen.

One of the localities that deserves particular attention is Lake Geneva (Lac Léman) on the Swiss-French border (marked by *L* in Fig. 1), where the signal crayfish has been intensively harvested [55]. In this lake, over 31% of analysed *P. leniusculus* individuals tested positive for *A. astaci*. Although French law does not permit the transport and selling of invasive species, special prefectoral regulations allow fishermen to sell crayfish from Lake Geneva anywhere in France. They may therefore be exported alive to the rest of the country under the label “crayfish from Lac Léman” [23], [55]. Such commercial activities may contribute to the spread of the crayfish plague. Indeed, one French aquaculture company who offers *P. leniusculus* from Lake Geneva also sells *A. leptodactylus* that do not seem to live long after purchase (T. Duperray, pers. comm.). More measures should therefore be taken to avoid the further spread of signal crayfish from this lake to other parts of France or beyond.

However, Lake Geneva is also interesting because of the evidence for the extended coexistence between American and European crayfish species. The signal crayfish has long been established in the lake, since 1976 [55]. However, the narrow-clawed crayfish *Astacus leptodactylus* (a European species not indigenous to this region) was found in the lake at least until 2001 [55] and it seems that the native white-clawed crayfish *Austropotamobius pallipes* was also present there at least until 2003 (C. Bugnon, pers. comm.). Several cases of long-term coexistence between native species and *P. leniusculus* have also been recorded in other European countries. In England, *A. pallipes* coexisted with signal crayfish for more than 5 years [4]. In Finland, Westman & Savolainen [56] reported a 30-year coexistence of *P. leniusculus* with another native species, *Astacus astacus*, although the latter was finally outcompeted. In these cases, it has been suggested that the invasive species was not infected by the pathogen, similarly as in some Central European sites where native crayfish coexisted with *O. limosus* [49]. The present situation in Lake Geneva, where we demonstrated the presence of *A. astaci* in five out of 16 analysed signal crayfish, seems thus different, although we cannot rule out that the pathogen has been introduced to the local population of *P. leniusculus* only recently.

It is probable that the signal crayfish as well as the spiny-cheek crayfish *O. limosus*, also present in the lake [55], have contributed to the decline of the susceptible species *Astacus leptodactylus* and *Austropotamobius pallipes* at this locality. No information on mass mortalities of these species in the lake exist, so we cannot assess if this decline was caused by plague outbreaks, competition with invasive crayfish, other reasons, or a combination of multiple factors. Nevertheless, several recent studies have demonstrated that chronic *A. astaci* infections of generally susceptible crayfish may be possible at some localities, as was shown for *A. leptodactylus* in Turkey [52], [57] and Romania [37], [58], for *Astacus astacus* in Finland [59], [60], and most recently for *Austropotamobius torrentium* in Slovenia [61]. Kozubíková *et al.* [33] also reported from the Czech Republic that the native noble crayfish *A. astacus* coexisted for at least ten years with a *P. leniusculus* population in which a low *A. astaci* agent level (A2) was recently found in two out of 23 analysed individuals (although a recent confirmation of the pathogen presence does not necessarily mean the host population has been infected during the whole period of coexistence). The presence of the crayfish plague pathogen apparently does not always result in the complete disappearance of native species at a locality. Such coexistence seems facilitated both by an increased resistance of some populations or species of European crayfish to the pathogen and a reduced virulence of some *A. astaci* strains [62], [63], the relative contributions of these (and possibly other) factors probably differing from site to site.

In some areas in France, *Austropotamobius pallipes* is still relatively abundant (although the sites occupied by invasive American crayfish are already much more numerous than those with native species [23]). In other regions in the country, this native species is only rare or even absent (see Fig. 1 and [23]). The main centres of distribution are in central and south-eastern France, the former being also occupied by many signal crayfish populations [23]. At several sites in Rhône-Alpes and Auvergne (Rh-Al and Au in Fig. 1), regions where mortalities of *A. pallipes* occurred in the past (T. Duperray, T. Pantarotto, pers. comm.), we found that nearby signal crayfish populations had individuals infected by *A. astaci*. High priority should therefore especially be given to the protection of those localities where *A. pallipes* is abundant but infected *P. leniusculus* populations are nearby.

Apart from screening for the presence of the pathogen, molecular methods may also be used to assess the pathways and likely sources of *Aphanomyces astaci.* We have directly demonstrated the pathogen by real-time PCR detection in samples of *A. pallipes* from two crayfish plague outbreaks from France (indicated by thicker red crosses in Fig. 1). The first took place in 2001 in the Saint-Christophe river, Poitou-Charentes region, Western France (Po-Ch in Fig. 1), and was described in detail in [46]. The second outbreak took place in July 2008 in the river Le Jabron, Rhône-Alpes region (Rh-Al in Fig. 1). In both regions, all three widespread North American crayfish species (*Pacifastacus leniusculus*, *Orconectes limosus*, and *Procambarus clarkii*) are present [23], so any of them might have been the source of the pathogen causing these mass mortalities. However, as different genotypes of *A. astaci* have been isolated from different host species [64], [65], genotyping of the pathogen might indicate its source. We characterised *A. astaci* from both French outbreaks using several variable microsatellite markers that are being developed for this species (F. Grandjean et al., unpublished), and confirmed that the first mortality was caused by the genotype group B, which is associated with *P. leniusculus* [64], and the other one by the genotype group E, originally isolated from *O. limosus* [65]. Although the reliability of the pathogen genotyping approach needs thorough evaluation, these results indeed suggest that signal crayfish has been the original source of *A. astaci* in at least some mass mortalities recently recorded in France.

The trend of increase in crayfish plague prevalence and the pathogen load of local infected individuals in French *P. leniusculus* populations corresponds to findings of Kozubíková *et al.* [33], who observed similar but much more pronounced patterns in Central European *O. limosus* and *P. leniusculus* populations. This relationship might be due to increased concentrations of *A. astaci* zoospores in the environment released from infected individuals, increasing the likelihood of pathogen spread. However, the pattern observed in French signal crayfish populations studied by us was not significant, unlike those reported in [33].

Eradication of *P. leniusculus* from large areas is not yet possible [4]. However, our results can contribute to the development of more efficient conservation management strategies for native crayfish in France. More attention should be paid to areas where high prevalences of the crayfish plague in tested *P. leniusculus* populations have been found, as these represent a greater danger to native species. The fact that we found infected individuals in more than half of the studied populations confirms that the signal crayfish plays an important role in the transmission of the crayfish plague pathogen in France, and that it represents a serious threat to native crayfish, especially to the endangered *Austropotamobius pallipes*. Our analysis also confirms that other non-indigenous crayfish species, including the recently established invader *Orconectes immunis,* likely serve as sources of the disease in France. Further studies of the prevalence of the crayfish plague in other non-indigenous host species would therefore also be important for evaluating the risk they represent for native crayfish.

## References

[1] Holdich DM (2003) Crayfish in Europe – an overview of taxonomy, legislation, distribution, and crayfish plague outbreaks. In: Holdich DM, Sibley PJ, editors. Management and Conservation of Crayfish. Proceedings of a conference held on 7th November 2002 at the Nottingham Forest Football Club, Nottingham, UK. Bristol: Environment Agency. 15–34.

[2] Alderman DJ (1996) Geographical spread of bacterial and fungal diseases of crustaceans. Rev Sci Tech - Off Int Epizoot 15: 603–632. 10.20506/rst.15.2.943 8890383

[3] Holdich DM (2002) Distribution of crayfish in Europe and some adjoining countries. Bull Fr Pêche Piscic 367: 611–650.

[4] Souty-Grosset C, Holdich DM, Noël PY, Reynolds JD, Haffner P (2006) Atlas of crayfish in Europe. Patrimoines naturels, 64. Paris: Muséum national d’Histoire naturelle. 187 p.

[5] Holdich DM, Reynolds JD, Souty-Grosset C, Sibley PJ (2009) A review of the ever increasing threat to European crayfish from non-indigenous crayfish species. Knowl Manag Aquat Ecosyst 394–395: 11.

[6] Vennerström P, Söderhäll K, Cerenius L (1998) The origin of two crayfish plague (*Aphanomyces astaci*) epizootics in Finland on noble crayfish, *Astacus astacus* . Ann Zool Fennici 35: 43–46.

[7] Oidtmann B, Cerenius L, Schmid I, Hoffmann R, Söderhäll K (1999) Crayfish plague epizootics in Germany – classification of two German isolates of the crayfish plague fungus *Aphanomyces astaci* by random amplification of polymorphic DNA. Dis Aquat Organ 35: 235–238.

[8] Kozubíková E, Petrusek A, Ďuriš Z, Martín MP, Diéguez-Uribeondo J, et al.. (2008) The old menace is back: recent crayfish plague outbreaks in the Czech Republic. Aquaculture, 274, 208–217.

[9] Raveret-Wattel C (1885) Résumé des réponses au questionnaire sur la maladie des écrevisses [Summary of responses on a questionnaire concerning crayfish disease]. Bull Soc Nat d’ Acclimat de France, Paris 2: 614–633 (in French)..

[10] Machino Y, Diéguez-Uribeondo J (1998) Un cas de peste des écrevisses en France dans le bassin de la Seine [Example of the crayfish plague presence in France in the Seine watershed]. L’Astaciculteur de France 54: 2–11 (in French with English abstract)..

[11] Edgerton BF, Henttonen P, Jussila J, Mannonen A, Paasonen P, et al (2004) Understanding the causes of disease in European freshwater crayfish. Conserv Biol 18: 1466–1474.

[12] Collas M, Salek X (2002) Description d’un cas de peste ou Aphanomycose dans le département des Vosges [Description of an outbreak of crayfish plague or aphanomycosis in Vosges department]. L’Astaciculteur de France 70: 2–6 (in French with English abstract)..

[13] Neveu A (1998) *Pacifastacus leniusculus*: son rôle de vecteur et de réservoir de la peste des écrevisses (Aphanomycose). Etat actuel des connaissances [*Pacifastacus leniusculus* involved as a carrier and stock of the crayfish plague fungus: present knowledge]. L’Astaciculteur de France 57: 6–11 (in French with English abstract)..

[14] Neveu A (1998) Presence de l’aphanomycose en France: suivi d’un foyer dans l’ouest de 1990 à 1998. [Presence of aphanomycosis in France: survey of a focus in Western part of France from 1990 to 1998]. L’Astaciculteur de France 57: 2–6 (in French with English abstract)..

[15] Neveu A (2000) L’écrevisse de Louisiane (*Procambarus clarkii*): réservoir permanent et vecteur saisonnier de l’Aphanomycose dans un petit étang de l’ouest de la France [The red swamp crayfish (*Procambarus clarkii*): continuous reservoir and seasonal vector of crayfish plague in small ponds of the Western part of France]. L’Astaciculteur de France 63: 7–11 (in French with English abstract)..

[16] Neveu A (2002) *Pacifastacus leniusculus* Les espèces animales et végétales susceptibles de proliférer dans les milieux aquatiques et subaquatiques. Fiches - espèces animale*s* [Animal and plant species capable to proliferate in aquatic and submerged areas. Files – animal species]. Rapport de DESS, Agence de l’Eau Artois-Picardie (Douai). 87–91. (in French).

[17] Bohman P, Nordwall F, Edsman L (2006) The effect of the large-scale introduction of signal crayfish on the spread of crayfish plague in Sweden. Bull Fr Pêche Piscic 380–381: 1291–1302.

[18] Diéguez-Uribeondo J (2006) The dispersion of the *Aphanomyces astaci*-carrier *Pacifastacus leniusculus* by humans represents the main cause of disappearance of the indigenous crayfish *Austropotamobius pallipes* in Navarra. Bull Fr Pêche Piscic 380–381: 1303–1312.

[19] Arrignon JCV, Gépard P, Krier A, Laurent PJ (1999) The situation in Belgium, France, and Luxembourg. In: Gherardi F, Holdich DM, editors. Crayfish in Europe as alien species. How to make the best of a bad situation? Rotterdam, Brookfield: A.A. Balkema. 129–140.

[20] Grandjean F, Souty-Grosset C (1997) Preliminary results on the genetic variability of mitochondrial DNA in the signal crayfish *Pacifastacus leniusculus* Dana. C R Acad Sci III 320: 551–556. 9309256 10.1016/s0764-4469(97)84710-5

[21] Vigneux E (1997) Les introductions de crustacés décapodes d’eau douce en France. Peut-on parler de gestion? [Introductions of freshwater decapod crustaceans into France. Can we speak of management?]. Bull Fr Pêche Piscic 344–345: 357–370 (in French with English abstract)..

[22] Machino Y (1999) Introductions clandestines de *Pacifastacus leniusculus* dans la région Rhône-Alpes [Clandestine introductions of *Pacifastacus leniusculus* in the Rhône-Alpes Region]. L’Astaciculteur de France 60: 2–4 (in French with English abstract)..

[23] Collas M, Julien C, Monnier D (2007) La situation des écrevisses en France. Résultats des enquêtes nationales réalisées entre 1977 et 2006 par le Conseil Supérieur de la pêche [Situation of the crayfish in France. Results of the national surveys performed between 1977 and 2006 by the Conseil Supérieur de la Pêche (CSP)]. Bull Fr Pêche Piscic 386: 1–38 (in French with English abstract)..

[24] Bramard M, Demers A, Trouilhe M-C, Bachelier E, Dumas J-C, et al (2006) Distribution of indigenous and non-indigenous crayfish populations in the Poitou-Charentes region (France): evolution over the past 25 years. Bull Fr Pêche Piscic 380–381: 857–866.

[25] Alderman DJ, Polglase JL (1986) *Aphanomyces astaci*: isolation and culture. J Fish Dis 9: 367–379.

[26] Oidtmann B, Bausewein S, Hölzle L, Hoffmann R, Wittenbrink M (2002) Identification of the crayfish plague fungus *Aphanomyces astaci* by polymerase chain reaction and restriction enzyme analysis. Vet Microbiol 85: 183–194. 11844624 10.1016/s0378-1135(01)00505-3

[27] Oidtmann B, Schaefers N, Cerenius L, Söderhäll K, Hoffmann RW (2004) Detection of genomic DNA of the crayfish plague fungus *Aphanomyces astaci* (Oomycete) in clinical samples by PCR. Vet Microbiol 100: 269–282. 15145505 10.1016/j.vetmic.2004.01.019

[28] Oidtmann B, Geiger S, Steinbauer P, Culas A, Hoffmann RW (2006) Detection of *Aphanomyces astaci* in North American crayfish by polymerase chain reaction. Dis Aquat Organ 72: 53–64. 17067073 10.3354/dao072053

[29] Hochwimmer G, Tober R, Bibars-Reiter R, Licek E, Steinborn R (2009) Identification of two GH18 chitinase family genes and their use as targets for detection of the crayfish-plague oomycete *Aphanomyces astaci* . BMC Microbiology 9: 184. 19719847 10.1186/1471-2180-9-184 PMC2751781

[30] Vrålstad T, Knutsen AK, Tengs T, Holst-Jensen A (2009) A quantitative TaqMan MGB real-time polymerase chain reaction based assay for detection of the causative agent of crayfish plague *Aphanomyces astaci* . Vet Microbiol 137: 146–155. 19201113 10.1016/j.vetmic.2008.12.022

[31] Makkonen J, Jussila J, Kokko H (2012) The diversity of the pathogenic Oomycete (*Aphanomyces astaci*) chitinase genes within the genotypes indicate adaptation to its hosts. Fungal Genet Biol 49: 635–642. 22683546 10.1016/j.fgb.2012.05.014

[32] Skov C, Aarestrup K, Sivebæk F, Pedersen S, Vrålstad T, et al (2011) Non-indigenous signal crayfish *Pacifastacus leniusculus* are now common in Danish streams: preliminary status for national distribution and protective actions. Biol Invasions 13: 1269–1274.

[33] Kozubíková E, Vrålstad T, Filipová L, Petrusek A (2011) Re-examination of the prevalence of *Aphanomyces astaci* in North American crayfish populations in Central Europe by TaqMan MGB real-time PCR. Dis Aquat Organ 97: 113–125. 22303628 10.3354/dao02411

[34] Kozubíková E, Filipová L, Kozák P, Ďuriš Z, Martín MP, et al (2009) Prevalence of the crayfish plague pathogen *Aphanomyces astaci* in invasive American crayfishes in the Czech Republic. Conserv Biol 23: 1204–1213. 19459897 10.1111/j.1523-1739.2009.01240.x

[35] Johnsen SI, Taugbøl T, Andersen O, Museth J, Vrålstad T (2007) The first record of the non-indigenous signal crayfish *Pasifastacus leniusculus* in Norway. Biol Invasions 9: 939–941.

[36] Vrålstad T, Johnsen SI, Fristad RF, Edsman L, Strand D (2011) Potent infection reservoir of crayfish plague now permanently established in Norway. Dis Aquat Organ 97: 75–83. 22235597 10.3354/dao02386

[37] Pârvulescu L, Schrimpf A, Kozubíková E, Cabanillas Resino S, Vrålstad T, et al (2012) Invasive crayfish and crayfish plague on the move: first detection of the plague agent *Aphanomyces astaci* in the Romanian Danube. Dis Aquat Organ 98: 85–94. 22422132 10.3354/dao02432

[38] Tuffs S, Oidtmann B (2011) A comparative study of molecular diagnostic methods designed to detect the crayfish plague pathogen, *Aphanomyces astaci* . Vet Microbiol 153: 343–353. 21763084 10.1016/j.vetmic.2011.06.012

[39] Peay S (2009) Selection criteria for “ark sites” for white-clawed crayfish. In: Brickland J, Holdich DM, Imhoff EM, editors. Crayfish Conservation in the British Isles 2009. Proceedings of a conference held on 25th March 2009 in Leeds, UK. 63–69.

[40] Söderhäll K, Cerenius L (1998) Role of the prophenoloxidase-activating system in invertebrate immunity. Curr Opin Immunol 10: 23–28. 9523106 10.1016/s0952-7915(98)80026-5

[41] Nylund V, Westman K (1983) Frequency of the visible symptoms of crayfish plague fungus *Aphanomyces astaci* on the American crayfish *Pacifastacus leniusculus* in natural populations in Finland. Freshw Crayfish 5: 277–283.

[42] Nylund V, Westman K (2000) The prevalence of crayfish plague (*Aphanomyces astaci*) in two signal crayfish (*Pacifastacus leniusculus*) populations in Finland. J Crustacean Biol 20: 777–785.

[43] Strand DA, Holst-Jensen A, Viljugrein H, Edvardsen B, Klaveness D, et al (2011) Detection and quantification of the crayfish plague agent in natural waters: direct monitoring approach for aquatic environments. Dis Aquat Organ 95: 9–17. 21797031 10.3354/dao02334

[44] Filipová L (2012) Genetic variation in North American crayfish species introduced to Europe and the prevalence of the crayfish plague pathogen in their populations. Ph.D. thesis. Prague: Charles University in Prague & Poitiers: Université de Poitiers. 132 p.

[45] Papin JC (2000) Mon astaciculture de pieds rouges et de pattes grêles ruinée, forte présomption d’aphanomycose [My crayfish farm of noble crayfish and narrow clawed crayfish destroyed: crayfish plague strongly suspected]. L’Astaciculteur de France 63: 11–12 (in French with English abstract)..

[46] Neveu A, Bachelier E (2002) Mortalité d’*Austropotamobius pallipes* sur le bassin de la Sèvre Niortaise. Présence de l’Aphanomycose. [*Austropotamobius pallipes* mortality in the Sèvre Niortaise river system. Presence of plague disease (aphanomycosis)]. L’Astaciculteur de France 76: 2–4 (in French with English abstract)..

[47] Stevenson M, Nunes T, Sanchez J, Thornton R, Reiczigel J, et al.. (2013) epiR: An R package for the analysis of epidemiological data. R package version 0.9–48. http://CRAN.R-project.org/package=epiR.

[48] R Core Team (2013) R: A language and environment for statistical computing. R Foundation for Statistical Computing, Vienna, Austria.

[49] Schrimpf A, Maiwald T, Vrålstad T, Schulz HK, Śmietana P, et al (2013) Absence of the crayfish plague pathogen (*Aphanomyces astaci*) facilitates coexistence of European and American crayfish in central Europe. Freshw Biol 58: 1116–1125.

[50] Schrimpf A, Chucholl C, Schmidt T, Schulz R (2013) Crayfish plague agent detected in populations of the invasive North American crayfish *Orconectes immunis* (Hagen, 1870) in the Rhine River, Germany. Aquat Invasions 8: 103–109.

[51] Skov C, Sivebæk F, Aarestrup K, Vrålstad T, Hansen PG, et al.. (2009) Udbredelse og bekæmpelse af signalkrebs i Alling Å [Distribution and tools to potential eradication of signal crayfish in river Alling]. Report from DTU Aqua to the County of Randers and the Danish Forest and Nature Agency. 39 p. (in Danish).

[52] Makkonen J, Kokko H, Henttonen P, Kivistik M, Hurt M, et al (2010) Fungal isolations from Saaremaa, Estonia: Noble crayfish (*Astacus astacus*) with melanised spots. Freshw Crayfish 17: 155–158.

[53] Svoboda J, Kozubíková E, Kozák P, Kouba A, Bahadir Koca S, et al (2012) PCR detection of the crayfish plague pathogen in narrow-clawed crayfish inhabiting Lake Eğirdir in Turkey. Dis Aquat Organ 98: 255–259. 22535876 10.3354/dao02445

[54] Matasová K, Kozubíková E, Svoboda J, Jarošík V, Petrusek A (2011) Temporal variation in the prevalence of the crayfish plague pathogen, *Aphanomyces astaci*, in three Czech spiny-cheek crayfish populations. Knowl Manag Aquat Ecosyst 401: 14.

[55] Dubois J-P, Gillet C, Laurent PJ, Michoud M (2003) Que sont devenues les populations d’écrevisses de la rive francaise du Lac Leman? [What happened to the crayfish populations of the French shore of the lake Geneva? ] L’Astaciculteur de France 77: 2–11 (in French with English abstract)..

[56] Westman K, Savolainen R (2001) Long term study of competition between two co-occurring crayfish species, the native *Astacus astacus* L. and the introduced *Pacifastacus leniusculus* Dana, in a Finnish lake. Bull Fr Pêche Piscic 361: 613–627.

[57] Kokko H, Koistinen L, Harlıoğlu MM, Makkonen J, Aydın H, et al (2012) Recovering Turkish narrow clawed crayfish (*Astacus leptodactylus*) populations carry *Aphanomyces astaci* . Knowl Manag Aquat Ecosyst 404: 12.

[58] Schrimpf A, Pârvulescu L, Copilaş-Ciocianu D, Petrusek A, Schulz R (2012) Crayfish plague pathogen detected in the Danube Delta – a potential threat to freshwater biodiversity in southeastern Europe. Aquat Invasions 7: 503–510.

[59] Jussila J, Makkonen J, Vainikka A, Kortet R, Kokko H (2011) Latent crayfish plague (*Aphanomyces astaci*) infection in a robust wild noble crayfish (*Astacus astacus*) population. Aquaculture 321: 17–20.

[60] Viljamaa-Dirks S, Heinikainen S, Nieminen M, Vennerström P, Pelkonen S (2011) Persistent infection by crayfish plague *Aphanomyces astaci* in a noble crayfish population – a case report. Bull Eur Assoc Fish Pathol 31: 182–188.

[61] Kušar D, Vrezec A, Ocepek M, Jenčič V (2013) Crayfish plague (*Aphanomyces astaci*) in wild crayfish populations in Slovenia: first report of persistent infection in stone crayfish *Austropotamobius torrentium* population. Dis Aquat Organ 103: 157–169. 23548366 10.3354/dao02567

[62] Makkonen J, Jussila J, Kortet R, Vainikka A, Kokko H (2012) Differing virulence of *Aphanomyces astaci* isolates and elevated resistance of noble crayfish *Astacus astacus* against crayfish plague. Dis Aquat Organ 102: 129–136. 23269387 10.3354/dao02547

[63] Viljamaa-Dirks S, Heinikainen S, Torssonen H, Pursiainen M, Mattila J, et al (2013) Distribution and epidemiology of the crayfish plague agent *Aphanomyces astaci* genotypes from noble crayfish *Astacus astacus* in Finland. Dis Aquat Organ 103: 199–208. 23574706 10.3354/dao02575

[64] Huang TS, Cerenius L, Söderhäll K (1994) Analysis of genetic diversity in the crayfish plague fungus, *Aphanomyces astaci*, by random amplification of polymorphic DNA. Aquaculture 126: 1–9.

[65] Kozubíková E, Viljamaa-Dirks S, Heinikainen S, Petrusek A (2011) Spiny-cheek crayfish *Orconectes limosus* carry a novel genotype of the crayfish plague agent *Aphanomyces astaci* . J Invertebr Pathol 108: 214–216. 21856310 10.1016/j.jip.2011.08.002

